# Aspects épidémiologiques, cliniques et étiologiques des douleurs thoraciques en consultation externe de cardiologie à Ouagadougou

**DOI:** 10.11604/pamj.2014.19.260.5184

**Published:** 2014-11-08

**Authors:** Nobila Valentin Yaméogo, Larissa Justine Kagambèga, Aimé Arsène Yaméogo, Koudougou Jonas Kologo, Georges Rosario Christian Millogo, Boubacar Jean Yves Toguyéni, André Samadoulougou, Jacques Simporé, Patrice Zabsonré

**Affiliations:** 1 Service de Cardiologie, CHU Yalgado Ouédraogo, Ouagadougou, Burkina Faso; 2 Service de Cardiologie, CHU Sanou Souro, Bobo Dioulasso, Burkina Faso; 3 Centre de Recherche Biomoléculaire, Ouagadougou, Burkina Faso

**Keywords:** Douleur thoracique, consultation externe, cardiologie, épidémiologie, chest pain, outpatient consultation, cardiology, epidemiology

## Abstract

**Introduction:**

La douleur thoracique est un motif fréquent de consultation en cardiologie à tous les âges. L'objectif de ce travail était de décrire les aspects épidémiologique, clinique et étiologique des douleurs thoraciques en consultation externe de cardiologie.

**Methods:**

Il s'est agi d'une étude transversale descriptive réalisée dans deux hôpitaux de la ville de Ouagadougou. Nous avons enregistré les patients reçus en consultation externe de cardiologie pour douleur thoracique. Lorsque l'examen clinique et le bilan paraclinique plausible étaient non contributifs, la douleur était déclarée sans étiologie. Les données ont été analysées à l'aide du logiciel SPSS version 17.

**Results:**

Durant la période de l’étude, 25664 consultations ont été réalisées. La douleur thoracique était motif de consultation dans 19,7% des cas. L’âge moyen des patients consultant pour douleur thoracique était de 44,4 ± 16,2 ans (extrêmes de 14 et 78 ans) avec un sex-ratio de 1,08 en faveur des femmes. Le bulletin de consultation en cardiologie avait été rédigé par un médecin dans 29,4% des cas. Les douleurs thoraciques étaient à prédominance retrosternale dans 28,7% des cas, épigastrique dans 23,9% des cas et basithoracique dans 13,8% des cas. L'intensité était jugée faible dans 73,6% des cas. Il s'agissait essentiellement de douleur à type de brûlure dans 40,5% des cas. Les étiologies étaient dominées par les causes cardiaques (37,6%) et digestives (31,9%). Parmi les étiologies cardiaques les valvulopathies rhumatismales (34,4%), le rhumatisme articulaire aigu (24,9%) et l'insuffisance coronarienne (19,5%) étaient les plus fréquentes.

**Conclusion:**

Les douleurs thoraciques sont fréquentes en consultation externe de cardiologie. Les étiologies sont surtout cardiaques et digestives. Les étiologies cardiaques sont dominées par la pathologie rhumatismale. Les étiologies extracardiaques sont nombreuses. Ce constat est probablement lié aux consultations directes des patients et aux orientations faites par le personnel paramédical.

## Introduction

Les douleurs thoraciques constituent un motif fréquent de consultation en cardiologie en Europe [1, 2] mais aussi dans les pays en développement. La crainte d’être cardiaque motive parfois une consultation directe en cardiologie. La littérature scientifique médicale africaine subsaharienne fait très peu mention du poids de la douleur thoracique en consultation externe de cardiologie. La plus part des travaux s'appesantissent sur la douleur thoracique aux urgences [1]. C'est fort de ce constat que nous avons réalisé ce travail dans le but de décrire les aspects épidémiologique, clinique et étiologique des douleurs thoraciques en consultation externe de cardiologie à Ouagadougou (Burkina Faso).

## Méthodes

Du 1^er^ janvier au 31 décembre 2011, nous avons systématiquement enregistré de manière consécutive, tous les patients qui ont consulté en cardiologie et avons retenu pour l’étude tous les patients dont le motif de consultation était une douleur thoracique. Le travail a été mené dans 2 centres médicaux de la ville de Ouagadougou à savoir l'unité de consultation externe du service de cardiologie du CHU-Yalgado Ouédraogo et celle du Centre Médical Saint Camille. Les patients ont invariablement bénéficié d'un examen clinique complet. Nous avons recherché des caractéristiques d'orientation étiologique de la douleur, les facteurs de risque cardio-vasculaire et la provenance de la demande de soin (le patient lui-même ou un agent de santé). Quant au bilan paraclinique, la NFS, l'ionogramme sanguin, la vitesse de sédimentation, la C-Réactive Protéine, la créatininémie, l'uricémie, la glycémie, l’électrocardiogramme de surface, la radiographie téléthorax de face, et l’écho Doppler cardiaque étaient systématiques. Par contre, les D-dimères, la troponinémie, la fibroscopie digestive haute, l’épreuve d'effort et l’échographie abdominale étaient réalisés lorsque les caractéristiques de la douleur indiquaient leur réalisation. Au demeurant, les examens à visée étiologique étaient tous réalisés et leur négativité déterminait l'absence d’étiologie. Les données ont été analysées à l'aide du logiciel SPSS version 17. Les résultats ont été exprimés en moyenne et en pourcentage.

## Résultats

Durant la période de l’étude, 25664 consultations ont été réalisées dont 23040 (89,8%) au CHU-Yalgado Ouédraogo et 2624 (10,2%) au Centre Médical Saint Camille. La douleur thoracique constituait le motif de consultation dans 19,7% des cas, soit 19% des cas (4378 patients) au CHU-Yalgado Ouédraogo et 26% des cas (683 patients) au Centre Médical Saint Camille.

### Caractéristiques générales des patients qui ont consulté pour douleur thoracique

**Figure 1:**
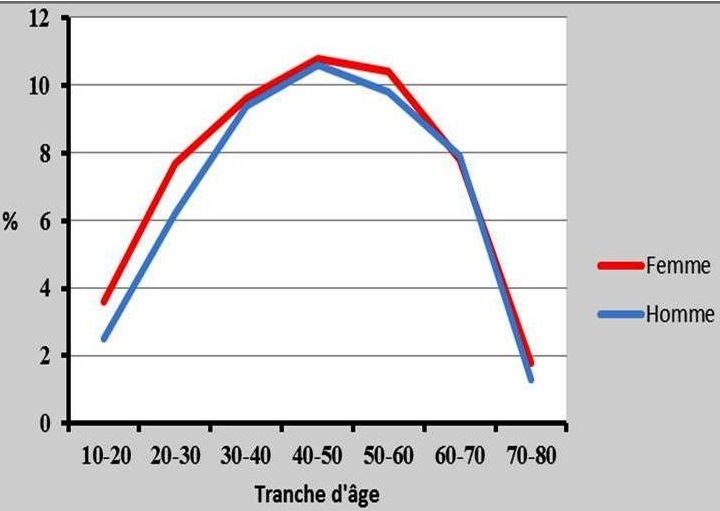
Fréquence des consultations en fonction du sexe et de l’âge de 5061 patients reçus en consultation externe de cardiologie pour douleur thoracique

La douleur thoracique représentait 19,7% des motifs de consultation. L’âge moyen des patients consultant pour douleur thoracique était de 44,4 ± 16,2 ans (extrêmes de 14 et 78 ans) avec un sex-ratio de 1,08 en faveur des femmes. Les sujets de moins de 40 ans représentaient 39,3% de l'effectif contre 60,7% pour les sujets d'au moins 40 ans. Plus de la moitié des patients venait de Ouagadougou et sa banlieue (69,2%). Les autres patients provenaient des autres provinces du Burkina. Le bulletin de consultation en cardiologie avait été rédigé par un infirmier dans 45,6% des cas et par un médecin dans 29,4% des cas. Dans les autres cas (25%) il s'agissait d'une demande directe des patients. Les tranches d’âge de 40 à 50 ans et 50 à 60 ans étaient les plus représentées. Le Tableau 1 résume la répartition de la population par tranche d’âge. Les facteurs de risque cardio-vasculaire étaient dominés par l’âge avancé (38,3%), l'hypertension artérielle (22,4%), la surcharge pondérale et l'obésité (8,6%) et la sédentarité (6,4%). Le Tableau 2 présente une synthèse des facteurs de risque cardio-vasculaire. Une hyperglycémie avait été retrouvée dans 7,1% des cas, une dyslipidémie dans 5,1% des cas et une hyper uricémie dans 5% des cas. Les paramètres biologiques anormaux des patients sont résumés dans le Tableau 3. La fréquence des consultations était sensiblement la même entre les hommes et les femmes quel que soit la tranche d’âge (Figure 1).

**Tableau 1:** Répartition de la population par tranche d’âge

Tranche d’âge	Population globale		Femme	Homme
	Nombre	%	%	%
[10-20[	313	6,2	3,6	2,5
[20-30[	709	14	7,7	6,3
[30-40[	967	19,1	9,7	9,4
[40-50[	1086	21,5	10,8	10,6
[50-60[	1024	20,2	10,4	9,8
[60-70[	801	15,8	7,9	7,9
[70-80[	162	3,2	1,8	1,4
total	5061	100	52	48

**Tableau 2:** Distribution des facteurs de risque cardio-vasculaire dans une population reçue pour une douleur thoracique en consultation externe de cardiologie

Facteur de risque	Nombre	Pourcentage
Age*	1939	38,3
Hypertension artérielle	567	22,4
Surcharge pondérale et obésité	435	8,6
Sédentarité	324	6,4
Diabète	273	5,4
Dyslipidémie	258	5,1
Evènement cardiovasculaire chez un parent de 1^er^ degré	167	3,3

* = (≥ 45 ans pour l'homme et 55 ans pour la femme)

**Tableau 3:** Paramètres biologiques anormaux des patients

Paramètre	Nombre	%
Hyperglycémie	359	7,1
Dyslipidémie	273	5,1
Hyper uricémie	253	5
Hypercréatininémie	207	4,1
Anémie	66	1,3
Augmentation de la CRP	54	1

### Caractères des douleurs thoraciques

Présentation clinique Les douleurs thoraciques étaient à prédominance retrosternale dans 28,7% des cas, épigastrique dans 23,9% des cas, basithoracique dans 13,8% des cas et latérothoraciques dans 11,4% des cas. Elles étaient diffuses dans 22,2% des cas. L'intensité était jugée faible dans 73,6% des cas, modéré dans 25,1% des cas et enfin intense dans 1,3% des cas. Il s'agissait essentiellement de douleur à type de brulure dans 40,5% des cas, de gêne dans 22,8% des cas, d'oppression dans 15,4% des cas, de picotement dans 13,4% des cas et de point de côté dans 7,9% des cas. Les aspects étiologiques Les étiologies étaient dominées par les étiologies cardiaques (37,6%) et digestives (31,9%). Le résumé de ces étiologies est représenté dans le Tableau 4. Parmi les étiologies cardiaques les valvulopathies rhumatismales (34,4%), le rhumatisme articulaire aigu (24,9%) et l'insuffisance coronarienne (19,5%) étaient les plus fréquentes. Le Tableau 5 résume les étiologies cardiaques des douleurs thoraciques.

**Tableau 4:** Distribution des étiologies des douleurs thoraciques chez 5061 patients reçus en consultation externe de cardiologie

Origine	Nombre	%
Cardiaque	1905	37,6
Digestive	1614	31,9
Pariétale	578	11,4
Pleuro-Pulmonaire	397	7,8
Autres étiologies	444	8,8
Sans étiologie	123	2,4
Total	5061	100

**Tableau 5:** Répartition des étiologies cardiaques des douleurs thoraciques de 5061 patients reçus en consultation externe de cardiologie

Etiologie	Nombre	%
Valvulopathie rhumatismale	655	34,4
Rhumatisme articulaire aigu	474	24,9
Insuffisance coronarienne	372	19,5
Cardiomyopathie dilatée	231	12,1
Péricardite	108	5,7
Trouble du rythme	83	4,3
Embolie pulmonaire	33	1,7

## Discussion

Les douleurs thoraciques constituent un motif fréquent de consultation en cardiologie. Mais la quasi-totalité des publications sur les douleurs thoraciques traite des douleurs aiguës. Notre étude a de ce fait le mérite de traiter des douleurs thoraciques en consultation externe de cardiologie. Dans notre étude, les douleurs thoraciques représentaient 19,7% des consultations. Une étude réalisée en Suisse en médecine de premier recours retrouvait la douleur thoracique comme motif de consultation dans 3,1% [1]. Il s'agit d'une étude réalisée dans un contexte de premier recours médical et non en milieu cardiologique. La différence de méthodologie entre nos études explique probablement cette différence de proportion des douleurs thoraciques. Les étiologies des douleurs thoraciques sont très variées [3]. Dans l’étude de Verdon [1] elles étaient dominées par les douleurs pariétales (51%). Dans notre étude par contre, les douleurs étaient d'origine cardiaque dans 37,6% des cas et digestives dans 31,9% des cas. La « douleur thoracique » est un des principaux motifs de recours au Samu et de prise en charge médicale préhospitalière. Selon Lapostolle et al., parmi les patients pris en charge pour une douleur thoracique en Seine-Saint-Denis au cours des dernières années, 300 par an environ présentaient une douleur thoracique liée à un syndrome coronaire aigu (SCA) avec sus-décalage du segment ST, environ 500 une douleur thoracique liée un SCA sans sus-décalage du segment ST et environ 800 une douleur thoracique d'une autre étiologie [4]. Ces autres diagnostics et/ou l'absence de signe de gravité permettent de différer l'enquête étiologique.

Selon la présomption étiologique de la douleur, le patient peut être orienté avec un niveau d'urgence variable, vers les services de spécialités. Dans les pays développés, la douleur thoracique aiguë est prise en charge par le SAMU avant l'admission du patient en unité de soins intensifs cardiologique [5, 6]. Dans ces pays, l'arrivée d'un patient présentant une douleur thoracique directement aux urgences est considérée comme un échec [4] en rapport avec un défaut d’éducation du patient [6, 7]. Dans notre système de santé, il n'existe pas de système d'appel. Tout patient formule sa demande de soin dans la structure sanitaire la plus proche qui décide d'orienter le patient s'il y a lieu. De nombreux patients sont orientés en cardiologie externe par le personnel paramédical. Les douleurs d'origine digestive occupent une place importante dans notre étude. Ceci est probablement dû au fait que nombre de patients sont orientés par le personnel paramédical et un nombre non négligeable consulte directement le cardiologue. Ainsi, il existe un flux important de consultation en cardiologie pour douleur thoracique, mais les douleurs non cardiaques sont très nombreuses. De ce fait le cardiologue se trouve submergé et doit réaliser un travail de triage.

## Conclusion

Les douleurs thoraciques sont fréquentes en consultation externe de cardiologie. Les étiologies sont essentiellement cardiaques et digestives. Les étiologies cardiaques sont surtout représentées par les valvulopathies rhumatismales et les poussées de rhumatisme articulaire aigu. Les étiologies non cardiaques sont nombreuses à cause d'une mauvaise orientation des malades ou des consultations cardiologiques directes. Il convient de réorganiser le système sanitaire afin d'assurer un triage convenable des patients afin que ne parviennent au cardiologue (ou autre spécialiste) les bonnes indications.
